# Modern Diseases

**Published:** 1889-10

**Authors:** 


### MODERN DISEASES.

It is a fact beyond dispute that the man of the present day is stronger, healthier, and less liable to attacks of disease, than in any preceding age. There are no more real diseases than there were a hundred years ago ; but, with our increasing knowledge, we are able to observe differences not then noted, and to separate into distinct diseases, each requiring different taeatment, certain affections which were formerly grouped together under one general name. Pneumonia, for instance, was rarely heard of in former times ; but "lung fever" was none the less fatal, and even more to be dreaded in the absence of any rational method of treatment. Before the general use of the microscope and chemical re-agents for testing the urine, diabetes, Bright's disease, and many others were not thoroughly differentiated, but were called by the general name of kidney trouble, and were just as common as in these modern times, when "safe" and infallible cures are advertised for sale at every drug store. The forms of brain disease accompanied by paralysis were in existence long before the term paresis was introduced into the medical vocabulary, and the victim of alcoholic dementia is not favored with the sight of any more zoological curiosities than his predecessor who merely had the common jam-jams.

"Heart failure" is the latest term of this sort employed to indicate the cause of death, and is a most unfortunate one. It really has no meaning at all, for the failure of the heart to do its work always occurs at the end of life. It may be said that death is always caused by the failure of either the heart or lungs to perform their duties, and that the various forms of accidents or disease are only indirect causes, inducing such failure. Heart failure is not a disease, but the result of disease, and there is nothing new about it whatever. When applied to organic or functional diseases of the heart it may have some significance ; but such a general term had best be used only in a very general sense.

The average length of life is greater, and the standard of bodily health higher than ever before ; and if it were not that medical and sanitary skill now preserves for a life of imperfect health many weak persons, who in former times would have succumbed to the first attack of disease, the standard would be even higher. In the works of old writers there are many passages which show that a man was considered old and past his prime at forty, while now at that age one is in the very height of his powers. There may be more diseases now than formerly ; but there is less disease, and a vastly greater knowledge of how to avoid it, or, when attacked, to bring it to a favorable termination.

